# Giant Left Atrial Myxoma Masquerading as Cough-Syncope Syndrome

**DOI:** 10.1177/2324709617724177

**Published:** 2017-08-04

**Authors:** Jennifer N. Bowman, Jennifer M. Treece, Pradnya Brijmohan Bhattad, Melania Bochis, Kailash Bajaj

**Affiliations:** 1 East Tennessee State University and James H. Quillen College of Medicine, Johnson City, TN, USA; 2 Grant Government Medical College and Sir J. J. Group of Hospitals, Mumbai, India

**Keywords:** Atrial myxoma, Syncopal episode, Cough-syncope syndrome

## Abstract

Left atrial myxomas are the most common type of benign primary cardiac tumor. Patients can present with generalized symptoms, such as fatigue, symptoms from obstruction of the myxoma, or even embolization of the myxoma causing distal thrombosis. We describe a case with several-month duration of syncopal episodes that occurred after coughing and with exertion. Computed tomography of the chest showed a 6.1 cm by 4.5 cm mass in the left atrium, later evaluated with an echocardiogram. Cardiothoracic surgery removed the mass, and it was determined to be an atrial myxoma. It is important for an internist to be able to diagnose an atrial myxoma because of the risks associated with embolization and even sudden death as myxoma can block blood supply from atrium to ventricle.

## Case Report

A 46-year-old male presented to the emergency department with several-month duration of exertional dyspnea after taking only a few steps with subsequent nonproductive coughing episodes leading to syncopal events. These episodes were ongoing over the past 8 months and had worsened over the 2 months prior to presentation. The patient was unable to work or drive for fear of coughing and suddenly losing consciousness. The patient also had 2-month duration of bilateral lower extremity edema, abdominal distension, and generalized fatigue with diagnoses of portal venous thrombosis at the same time, with anticoagulation noncompliance.

On presentation during this admission, the patient’s vital signs were within normal limits, and the patient was afebrile and had an oxygen saturation of 97% on room air. Physical exam was significant for an elevated jugular venous pressure, a loud S1, an early diastolic tumor plop but no murmurs, a positive hepatojugular reflux, and 1+ pedal edema in bilateral lower extremities. Lungs were clear to auscultation bilaterally. Pertinent laboratory work included a brain natriuretic peptide level of 1411 pg/mL and an erythrocyte sedimentation rate (ESR) of 12 mm/h.

**Figure 1.**
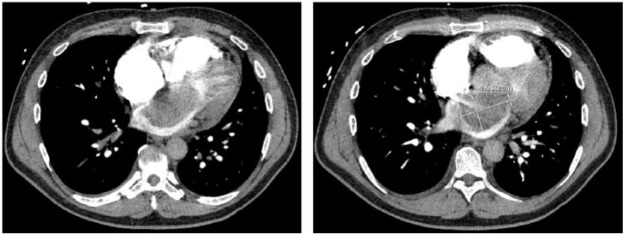
CT chest of a large left atrial myxoma. Dimensions of the LA mass are 6.1 cm × 4.5 cm. We could not determine whether the LA mass is a LA myxoma or a large thrombus on chest CT scan.

In the emergency room, the patient underwent a computed tomography (CT) of the chest (Figure 1), which demonstrated a 6.1 cm by 4.5 cm mass in the left atrium. A transthoracic echocardiogram with contrast was performed for further evaluation. Left ventricular ejection fraction was 60% to 65%, and the right ventricle was moderately dilated with moderate systolic dysfunction. The left atrium was moderately enlarged and a massive echodensity occupied the entire left atrium. Severe obstructive stenosis across the mitral valve was suggested by the mean transmitral gradient. The tricuspid valve showed moderate regurgitation with a jet velocity suggestive of severe pulmonary hypertension. Cardiac catheterization performed prior to surgery showed mild nonobstructive coronary artery disease.

**Figure 2.**
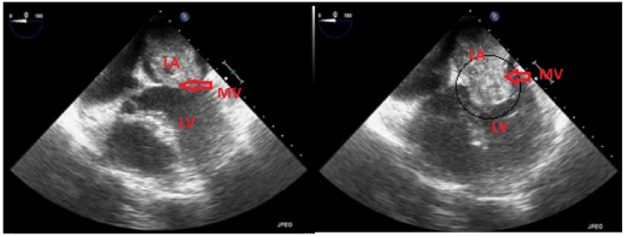
Transesophageal echocardiogram of the large obstructing left atrial myxoma. The dimensions of the LA myxoma were 6.1 cm × 4.5 cm and occupied most of the volume of the LA and caused severe obstructive stenosis across the mitral valve. The patient was also found to have severe pulmonary hypertension (LA, left atrium; LV, left ventricle; MV, mitral valve).

The patient underwent cardiothoracic surgery for removal of the left atrial mass, which was subsequently determined to be a left atrial myxoma. A transesophageal echocardiogram (Figure 2) was performed preoperatively and postoperatively. Preoperative findings included the left atrial mass, which appeared to be attached to the interatrial septum, biatrial enlargement, and a severely dilated and hypokinetic right ventricle. Postoperative findings showed complete removal of the mass and trivial mitral and tricuspid regurgitation. Histopathology confirmed the diagnosis of the cardiac myxoma.

The patient was discharged on postoperative day 3 with the following medications: rivaroxaban, losartan, metoprolol tartrate, and aspirin, and the patient was to follow-up with a primary care provided and continue taking rivaroxaban.

Hypercoagulability workup when not on anticoagulation was significant for a low protein C level. The patient was discharged home on long-term anticoagulation secondary to portal vein thrombosis with protein C deficiency.

## Epidemiology

Tumors that originate in the myocardium are rare, accounting for less than 0.1% of the population.^1^ Of the primary cardiac tumors, approximately 80% are considered benign, and atrial myxomas account for approximately half of the benign tumors.^2^ Papillary fibroelastomas and lipomas are other types of benign cardiac tumors in adults, and rhabdomyomas and fibromas are common pediatric cardiac tumors.^3^ Myxomas can vary in consistency but usually are composed of a scattering of cells with a mucopolysaccharide stroma.^4^ Approximately 80% to 90% of myxomas occur in the left atrium.^5,6^ The remainder of myxomas occur primarily in the right atrium with a very rare amount (less than 2%) occurring in the ventricles.^7^ Atrial myxomas are more common in females,^5,8^ but males are more likely to have embolization as a sequela of a myxoma.^9^

## Signs and Symptoms

The production of signs and symptoms from a myxoma is dependent on the location of the myxoma and include embolization, obstruction of blood flow through the heart, invasion of the tumor in surrounding myocardium and lung tissue, and constitutional symptoms. Myxomas that originate from the left atrium and the aortic valve are at the highest risk of embolization, which occur to the systemic circulation.^10^ In patients with a left atrial myxoma, symptoms may include signs of systemic embolization, such as cerebral emboli leading to neurological symptoms as well as emboli to other organs leading to myocardial infarction, renal failure, or mesenteric ischemia.^11^ Right atrial myxomas may lead to embolization into the pulmonic circulation leading to symptoms of pulmonary embolism. Left atrial tumors may also obstruct blood flow to the systemic circulation causing mitral valve regurgitation and heart failure signs and symptoms and can lead to secondary pulmonary hypertension.^5,12^ Myxomas may also lead to invasion of the myocardium or the adjacent lung leading to arrhythmias, pericardial effusion, or pulmonary symptoms.^12,13^ Constitutional symptoms such as fatigue and weight loss are common in 34% of patients.^14^

## Evaluation

A cardiac tumor may be visualized on CT scan of the chest, but it is difficult to differentiate a cardiac mass versus thrombus. Transthoracic echocardiography usually identifies the mass, but transesophageal echocardiography often provides better visualization of the left atrium and allows for clear differentiation of a tumor versus a thrombus by determining whether the mass has a stalk and therefore is a tumor or is unconnected from the intra-myocardial wall and therefore is a thombus.^15,16^ An echocardiogram also provides information about the mass including whether it obstructs circulation. Cardiac magnetic resonance imaging is another option for evaluating a cardiac tumor.^17^ Laboratory assessment includes an elevated ESR or C-reactive protein (CRP), which is thought to be due to an autoimmune component involving interleukin-6.^18,19^ Elevated ESR and CRP occurs in approximately 40% of patients with myxoma and are more common in patients with systemic symptoms.^11^

## Treatment

Surgical removal is the best treatment option if possible, as the risk of embolization is significant if the atrial myxoma is left in place. The most concerning consequence of an atrial myxoma is the risk of embolization and sudden death in up to one third of patients; therefore, urgent surgical resection of myxomas is recommended.^20-22^ Postoperative mortality after resection is generally low at less than 5%,^11^ and the 20-year survival rate is approximately 85%.^22^

## Risk of Recurrence

Risk of recurrence is approximately 5%^22^ and is often more common when the initial myxoma presentation is multicentric.^9^

## Discussion

The patient introduced in this case report had a unique presentation of a left atrial myxoma, although other cases of atrial myxomas discovered after syncopal episodes have also been reported in literature.^23-25^ Common presenting signs and symptoms include generalized symptoms like fatigue and weight loss, as well as cardiovascular symptoms. These cardiovascular symptoms come from mitral valve obstruction and/or elevated left atrial pressure and include chest pain, dyspnea on exertion, orthopnea, and paroxysmal nocturnal dyspnea.^11,25-27^ Other significant manifestations are neurological deficits from embolization of the myxoma, leading to events related to cerebral ischemia or infarction,^27,28^ retinal artery occlusion,^29^ and even sudden death.^21^ However, patients can also present asymptomatically or with minimal cardiac symptoms.^24^

**Figure 3.**
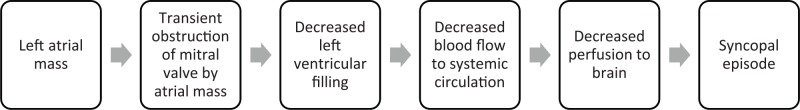
Syncopal episode physiology.

The patient in this case report endorsed generalized fatigue and symptoms of mitral valve obstruction including dyspnea on exertion, loud S1, and a tumor plop (ie, an early diastolic sound due to movement of the myxoma across the mitral valve into the left ventricle). The patient’s frequent syncopal episodes can be explained by the large left atrial mass obstructing the outflow of blood to the brain and thereby inducing syncopal events (see Figure 3). In addition, the mitral valve obstruction causes a sudden increase in left atrial pressure, causing the patient to cough.

The decision to provide anticoagulation to a patient after successful removal of a cardiac myxoma is not well studied. No clinical studies reviewing anticoagulation and long-term outcomes have been published to date. Existing case reports include anticoagulation when a patient initially presents with a pulmonary embolism (PE) and a right atrial myxoma, as a PE can reoccur in 0.4% to 5% of patients after right myxoma resection.^1,30^ Causes of recurrent PE following surgical excision of the myxoma include incomplete surgical resection of the myxoma and detachment of emboli during surgical removal.^1^ In general, a patient is at a higher risk of recurrent embolism if emboli are from multiple origins, if there is a family history of myxomas (eg, Carney complex), or if the myxoma reoccurs from progenitor cells.^1^ This is due to myxomatous tissue being typically friable and easily detachable with ability to adhere to other sites, promoting embolization.^1,31^ The Carney complex consists of unusual skin pigmentation (lentigines), multiple myxomas in the heart and throughout the body, and tumors in endocrine glands.^32^ It accounts for 7% to 10% of cardiac myxomas.^33^ With the Carney complex, the risk of recurrence of an atrial myxoma is as high as 25%, whereas normal recurrence of the atrial myxoma is generally 1% to 3%.^34^ The risk and clinical manifestations of embolization depend on which chamber the atrial myxoma resides. With left atrial myxomas, embolism occurs in 30% to 45% of patients, and usually to the brain or kidneys. With right atrial myxomas, embolism occurs in 10% of patients, and these can lead to PE.^1,34^

Regardless, left atrial myxoma can cause positional symptoms such as paroxysmal nocturnal dyspnea, typical cardiac murmur such as tumor plop, and can cause sudden cardiac death as the worst scenario.^35^ A conglomeration of the aforementioned clinical symptoms and signs should raise a high suspicion for early diagnosis of atrial myxoma.

## Conclusion

Diagnosis of an atrial myxoma must be considered when a patient is found to have cardiac mass and variable constitutional symptoms from outflow obstruction or embolization. While primary cardiac tumors are rare, myxomas are the most common type of cardiac tumor. Surgical removal is the best option to avoid embolization and even sudden death. Prognosis directly after surgery and long-term is good, and myxoma recurrence is generally low. The need for long-term anticoagulation is controversial and needs more research due to the rarity of this condition.
